# Benchmarking for healthy food stores: protocol for a randomised controlled trial with remote Aboriginal and Torres Strait Islander communities in Australia to enhance adoption of health-enabling store policy and practice

**DOI:** 10.1186/s12889-024-19277-0

**Published:** 2024-07-05

**Authors:** Julie Brimblecombe, Megan Ferguson, Emma McMahon, Bronwyn Fredericks, Nicole Turner, Christina Pollard, Louise Maple-Brown, Joanna Batstone, Leisa McCarthy, Eddie Miles, Khia De Silva, Adam Barnes, Mark Chatfield, Amanda Hill, Meaghan Christian, Emma van Burgel, Molly Fairweather, Anna Murison, Dickson Lukose, Surekha Gaikwad, Meron Lewis, Rebekah Clancy, Claire Santos, Kora Uhlmann, Sarah Funston, Laura Baddeley, Sally Tsekouras, Jaithri Ananthapavan, Gary Sacks, Amanda Lee

**Affiliations:** 1 Department of Nutrition, Dietetics and Food, Monash University, Melbourne, VIC 3168 Australia; 2 School of Public Health, The University of Queensland, Level 4 Public Health Building, Herston, Brisbane, QLD 4072 Australia; 3 Menzies School of Health Research, Charles Darwin University, Royal Darwin Hospital Campus, Darwin, NT Australia; 4 Office of the Deputy Vice-Chancellor, The University of Queensland, Herston, QLD 4006 Australia; 5 Indigenous Allied Health Australia, Alia House, Napier Cl, 2600 Deakin, ACT Australia; 6 Charles Perkins Centre, University of Sydney John Hopkins Dr, Camperdown, NSW 2050 Australia; 7 Faculty of Health Sciences, Curtin School of Population Health, Curtin University, Perth, WA Australia; 8 Monash Data Futures Institute, Monash University, Melbourne, VIC Australia; 9 Community First Development, 1/67 Townshend St, Phillip, ACT 2606 Australia; 10 Arnhem Land Progress Aboriginal Corporation, 70 O’Sullivan Cct, East Arm, NT 0822 Australia; 11 NT Health, Manunda Place, 38 Cavenagh Street, Darwin, NT 0800 Australia; 12 Outback Stores, 67 Pruen Rd, Berrimah, NT 0828 Australia; 13 Miwatj Health Aboriginal Corporation, Lot 1425 Arnhem Road, Nhulunbuy, NT Australia; 14 Population and Primary Health Care Outreach Team, NT Health, Casuarina Plaza, 258 Trower Road, Darwin, NT 0810 Australia; 15 Health and Wellbeing Queensland, 139 Coronation Drive, Milton, QLD 4064 Australia; 16 Katherine West Health Board Aboriginal Corporation, 38 First St, Katherine, NT 0850 Australia; 17 Deakin Health Economics, School of Health and Social Development, Faculty of Health, Deakin University, Institute for Health Transformation, Geelong, Australia; 18 Global Centre for Preventive Health and Nutrition, School of Health and Social Development, Faculty of Health, Deakin University, Institute for Health Transformation, Geelong, Australia

**Keywords:** Indigenous health, Continuous improvement, Health policy, Diet, Benchmarking, Implementation strategy

## Abstract

**Background:**

Aboriginal and Torres Strait Islander communities in remote Australia have initiated bold policies for health-enabling stores. Benchmarking, a data-driven and facilitated ‘audit and feedback’ with action planning process, provides a potential strategy to strengthen and scale health-enabling best-practice adoption by remote community store directors/owners. We aim to co-design a benchmarking model with five partner organisations and test its effectiveness with Aboriginal and Torres Strait Islander community stores in remote Australia.

**Methods:**

Study design is a pragmatic randomised controlled trial with consenting eligible stores (located in very remote Northern Territory (NT) of Australia, primary grocery store for an Aboriginal community, and serviced by a Nutrition Practitioner with a study partner organisation). The Benchmarking model is informed by research evidence, purpose-built best-practice audit and feedback tools, and co-designed with partner organisation and community representatives. The intervention comprises two full benchmarking cycles (one per year, 2022/23 and 2023/24) of assessment, feedback, action planning and action implementation. Assessment of stores includes i adoption status of 21 evidence-and industry-informed health-enabling policies for remote stores, ii implementation of health-enabling best-practice using a purpose-built Store Scout App, iii price of a standardised healthy diet using the Aboriginal and Torres Strait Islander Healthy Diets ASAP protocol; and, iv healthiness of food purchasing using sales data indicators. Partner organisations feedback reports and co-design action plans with stores. Control stores receive assessments and continue with usual retail practice. All stores provide weekly electronic sales data to assess the primary outcome, change in free sugars (g) to energy (MJ) from all food and drinks purchased, baseline (July-December 2021) vs July-December 2023.

**Discussion:**

We hypothesise that the benchmarking intervention can improve the adoption of health-enabling store policy and practice and reduce sales of unhealthy foods and drinks in remote community stores of Australia. This innovative research with remote Aboriginal and Torres Strait Islander communities can inform effective implementation strategies for healthy food retail more broadly.

**Trial registration:**

ACTRN12622000596707, Protocol version 1.

**Supplementary Information:**

The online version contains supplementary material available at 10.1186/s12889-024-19277-0.

## Contributions to the Literature

- Demonstrates what is possible in public health food retail research with established partner relationships and Aboriginal and Torres Strait Islander store directors driving change for their community
- Provides a trial protocol to address a knowledge gap on effective implementation strategies for evidence-based health-enabling policy and practice in the food retail sector
- Presents a novel intervention that benchmarks store businesses on best-practice policy, practice, price and purchasing metrics and uses a continuous improvement approach to support stores to  adopt best-practice 
- Demonstrates an approach to scale health-enabling best-practice in food retail

## Background

A unique food retail sector exists across Aboriginal and Torres Strait Islander communities in remote Australia. Over one-third of the approximate 230 stores are owned by the community and/or an Indigenous corporation [1]. Evidence is mounting of bold policy steps taken by communities to ensure their stores enable, rather than impede, health and wellbeing, despite the logistical challenges in providing a quality food supply in Australia’s most remote locations [2–8]. Aboriginal and Torres Strait Islander directors, elected by their communities to govern the community store, have increasingly exercised their power to direct policy to restrict the promotion of unhealthy food whilst promoting healthy food purchases [3–8]. Social purpose and a worldview that centres the collective, drives this unique store policy reform led by Aboriginal and Torres Strait Islander people. This is in the context of extraordinarily high rates of type 2 diabetes and related cardiometabolic chronic conditions, that to our knowledge, are among the highest in the world [9–11]. 

The health and wellbeing of society as a whole is a function of a healthy food environment [12]. Food environments across the globe have drastically altered in recent decades. Industrialisation of global and national food systems and disregard for the natural environment has enabled a growing population-level consumption of highly palatable ultra-processed foods and a concomitant rapid rise in the global burden of preventable chronic diseases [13, 14]. Many Indigenous Peoples have had their food systems irrevocably altered and/or destroyed, and with this bear a disproportionate burden of preventable chronic disease [15–17]. Indigenous Peoples continually fight to redress the systemic economic, social and political inequities associated with the structures of colonisation and marginalisation that underpin this burden [18, 19]. Improvements in diet through reduced consumption of unhealthy foods alongside increased consumption of healthy foods, could potentially prevent one in every five deaths globally [20, 21].

The 2011 report of the UN Special Rapporteur on the Right to Health submitted to the UN General Assembly recognised global food industry transnational corporations as “primary drivers of diet-related NCDs” [22]. UNICEF recently made specific recommendations for governments and the food retail industry to enact policy to restrict the promotion of unhealthy foods, particularly ultra-processed foods, recognising their detrimental impact on human and planetary health [23]. In remote Australia, Aboriginal and Torres Strait Islander community stores partnered with researchers in 2018, to generate world-first evidence that retail restriction on placement and promotion of unhealthy foods could reduce unhealthy food sales with no adverse impact on business outcomes [4]. The 7 component strategy tested included no promotion of unhealthy food and drinks, no placement of unhealthy food and drinks in high traffic areas, reduced shelf facings of targeted products (table sugar, sugar-sweetened beverages, confectionery and sweet biscuits) and no refrigerated sugar-sweetened beverages > 600 ml). In 2022, the UK Government implemented legislation to restrict the promotion of high fat, sugar and salt (HFSS) products in prominent store locations, “making a solid start towards creating healthier retail outlets for consumers” [24, 25].

Whilst there is mounting evidence to inform best-practice policy for healthy food retail, an evidence gap exists on how to implement and sustain best-practice in the food retail sector, where there is no government regulation on marketing of unhealthy food in food retail. Audit with feedback is a common strategy used in health-care delivery to promote the implementation of evidence-based practice by health care professionals and their organisations [26]. It can provide objective data on discrepancies between practice and target performance, which can increase accountability of organisations to improve quality of care and thereby increase compliance with desired practice [26]. Feedback is more effective when accompanied by both explicit goals and an action plan [27]. Benchmarking, which uses methods of audit and feedback to consider performance against a comparator, such as a peer group and/or national standards, is now used by public health researchers to increase accountability and commitment of the food industry, including manufacturers and retailers, to implement best-evidence health practice [28–30].

The Access to Nutrition Initiative (ATNI) and The International Network for Food and Obesity/non-communicable diseases Research, Monitoring and Action Support (INFORMAS) benchmarked major international and Australian food and beverage companies on their nutrition-related policies [29, 31]. Some positive changes to internal food and beverage company policies, commitments, and disclosure practices related to obesity prevention and population nutrition were reported by BIA-Obesity Australia as a result of benchmarking [32]. INFORMAS has developed a set of health-enabling best-practice performance metrics to benchmark supermarkets and has applied these in Argentina, Australia, Ghana and New Zealand [33–36]. Little is known however about the application of benchmarking in smaller and remote store settings. Further there is little known on whether benchmarking with action plans can translate best-practice evidence into policy and practice change in the food retail setting.

Continuous quality improvement employs a Plan–Do–Study–Act cycle to gather data, assess performance and system support, and set goals and strategies needed to achieve change [37]. The National Framework for Continuous Quality Improvement in Primary Health Care for Aboriginal and Torres Strait Islander People 2018–2023 emphasises the importance of organisational commitment and whole team involvement to improve systems and health care delivery by continually encouraging teams to ask ‘How are we doing?’, ‘Can we do it better?’, ‘How will we know if it is better?’ [38]. A study with four remote Aboriginal communities in Australia showed that feedback on practice performance to community store directors and their store managers with action planning as part of a continuous improvement cycle, informed change in store practice that increased fruit and vegetable sales and reduced sugar sales [39, 40]. Whilst we have this evidence, an evaluation of remote store adoption of health-enabling best-practice found there was opportunity for best-practice evidence to be better integrated in to decision-making processes [41].

Benchmarking with continuous improvement therefore provides a potential strategy to strengthen health-enabling best-practice adoption by remote community store directors/owners. The goal of the research reported herein is to co-design a model to facilitate benchmarking in the remote store setting, and test its effectiveness. We hypothesise that a codesigned benchmarking intervention can improve the adoption of health-enabling store policy and practice and reduce sales of unhealthy foods and drinks and therefore free sugars to energy purchased, in remote community stores of Australia.

This paper outlines the study protocol for the Benchmarking for Healthy Remote Aboriginal and Torres Strait Islander stores randomised controlled trial. An assessment of adoptability (including an economic evaluation) will be conducted and described separately to this paper. We use the SPIRIT 2013 Checklist: Recommended items to address in a clinical trial protocol and related documents, for this purpose, and report our research practice against The Aboriginal and Torres Strait Islander Quality Appraisal Tool designed by the Centre of Research Excellence in Aboriginal Chronic Disease Knowledge Translation and Exchange (hereafter the ‘CREATE Tool’) [42, 43]. Developers of this tool encourage its use in planning research ‘to achieve appropriate, high quality and relevant research that benefits Aboriginal and Torres Strait Islander peoples’ [43]. It emphasises the importance of Aboriginal and Torres Strait Islander leadership and partnership and is designed so that research effort reflects Aboriginal and Torres Strait Islander Peoples’ ways of knowing, being, and doing [43].

## Methods

This is a store-level pragmatic randomised parallel group, 2-arm, superiority trial with a 1:1 allocation. The study commenced in 2022.

### Our partners

Extensive and respectful collaboration with stakeholders over 30 years has informed this protocol. Consultation occurred with representatives of Aboriginal Community Controlled Health Organisations (ACCHOs), remote food retail organisations, and government, including the Arnhem Land Progress Aboriginal Corporation (ALPA), Aboriginal Medical Services Association of the Northern Territory (AMSANT), Apunipima Cape York Health Council, Health and Wellbeing Queensland, Katherine West Health Board Aboriginal Corporation (Katherine West Health Board), Miwatj Health Aboriginal Corporation (Miwatj Health), National Indigenous Australians Agency (NIAA), Northern Territory Health (NT Health), Outback Stores Pty Ltd (Outback Stores), and Sunrise Health Service Aboriginal Corporation (Sunrise Health Service). ALPA, Katherine West Health Board, Miwatj Health, NT Health, Outback Stores, and Sunrise Health Service are partners in this research.

Partners have agreed to extend the scope of their nutrition practitioners to deliver the co-designed benchmarking model with remote community stores. This workforce is on the frontline of working with communities to improve food and nutrition [44]. Nutrition practitioners with ACCHOs and government, work across multiple remote communities sometimes in a dual clinical-public health nutrition role, and support health education-type activities with stores such as group/staff education, individual store assessment/support, and cooking demonstrations [45]. ALPA and Outback Stores nutritionists are directly engaged with remote store operations and policy-making. At the time of writing this paper, ALPA has three nutritionists (including their Nutrition Manager) and Outback Stores has one (Health and Nutrition Manager).

### Study design and setting

This research is conducted in very remote Australia [46]. Most of Australia’s 7 692 024 km^2^land mass is classified as very remote with over 230 communities and a majority population of Aboriginal and Torres Strait Islander Peoples [47, 1]. These communities have a population of mostly over 250 to 1000. There are approximately eight communities of over 1000 in population and four under 250 [47]. This does not include ‘homeland’ communities or outstations that are outside larger communities and defined by the residents' cultural or traditional relationship to the land [48]. Community services generally include a school, health service, arts and cultural centre and local government council that provides essential services, and aged-care and child-care services. Most communities have a community store that that is the primary source of food for the community, with some also providing an automated teller machine, fuel depot and/or post office service [49]. Many community stores provide pre-prepared take-away meals. Large-sized communities may have more than one retail food store; most communities however are serviced by one store where residents shop, with the next nearest store a number of hours’ drive away [50].

As of 2020 16.1% of the Australian land mass is Aboriginal and Torres Strait Islander owned under ‘Aboriginal freehold land’ title through *Aboriginal Land Rights 1976 (NT) Act (ARLA)* [51–53]. This primarily occurs in the Northern Territory with 47% of the NT land mass held under Aboriginal freehold land title giving Aboriginal People of the region full ownership and rights over their land to preserve for future generations, excluding resources [52, 53]. The rights granted as part of Aboriginal and Torres Strait Islander Native title and Freehold title include collection of traditional food including hunting and fishing [54–56].

Indigenous Peoples have the right to participate in decisions that impact their lives [57]. Decisions on the type of products made available to community residents and how they are priced and promoted in-store impact health and wellbeing [58]. These decisions may be the responsibility of Aboriginal and Torres Strait Islander directors elected by the community and with a legal requirement to meet quarterly, or the responsibility of private operators depending on the business type [2]. Stores across remote Australia may be owned by the community, be privately owned where the operator has a lease agreement with the community land-owners through a lands council, and/or be government owned [2]. Community store directors may self-manage (referred to herein as independent stores) and/or contract a management service (referred to herein as store group stores) that may manage singular or multiple stores [2].

### Participants, interventions and outcomes

#### Participant eligibility criteria—inclusion and exclusion criteria

**Fig. 1:**
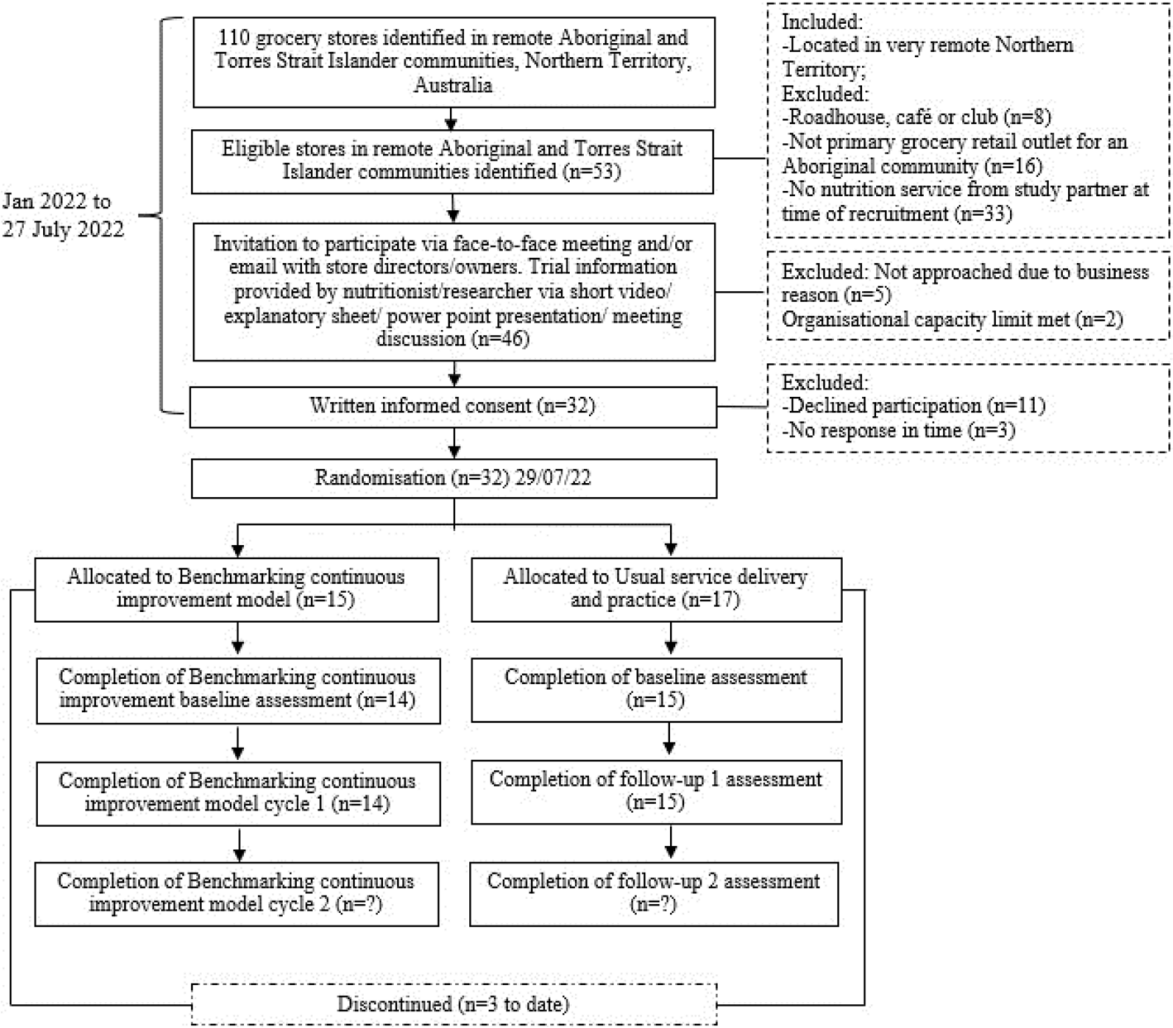
SPIRIT Participant Flow Diagram

Stores located in the very remote NT of Australia that are also a primary grocery retail outlet for an Aboriginal community, and serviced by a Nutrition Practitioner with a study partner were eligible (*n* = 53 of 110 in total). Store businesses were not eligible if they did not meet the inclusion criteria and/or are a roadhouse, cafe or club (i.e., not primarily a grocery store). See Fig. 1 for SPIRIT participant flow diagram.

#### Intervention

The Benchmarking model is informed by research evidence and the best-practice ‘audit and feedback’ tools our team purposely designed for use with remote community stores, and co-designed with partners and community through two workshops (2022 and 2025), a co-design committee which includes partner representatives and other experts and meets monthly, and task groups which meet regularly [59–62].

**Fig. 2:**
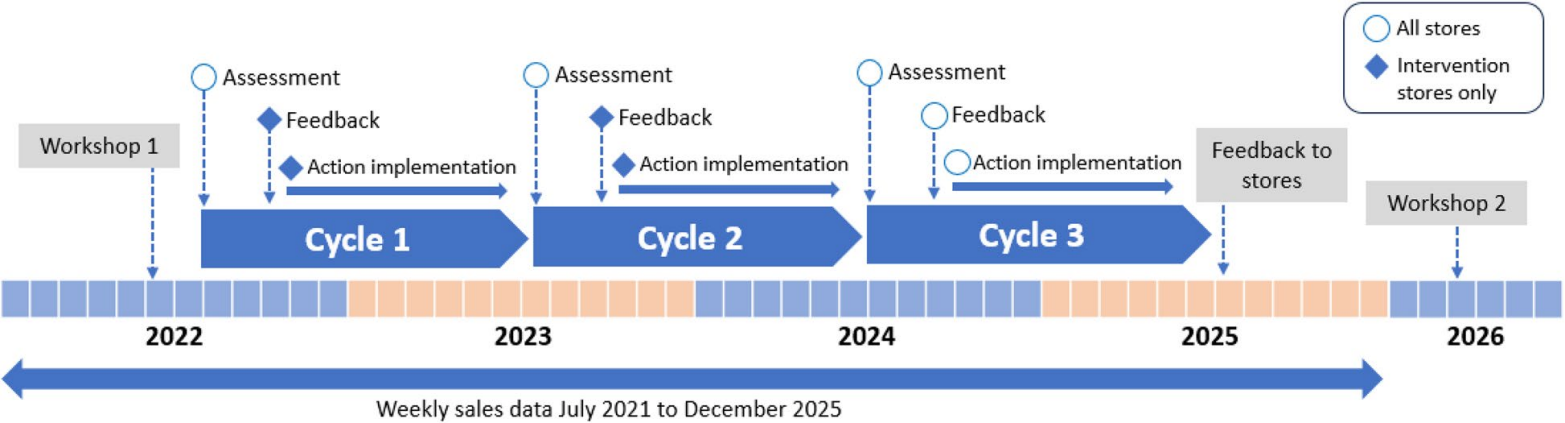
Timeline of intervention

**Fig. 3:**
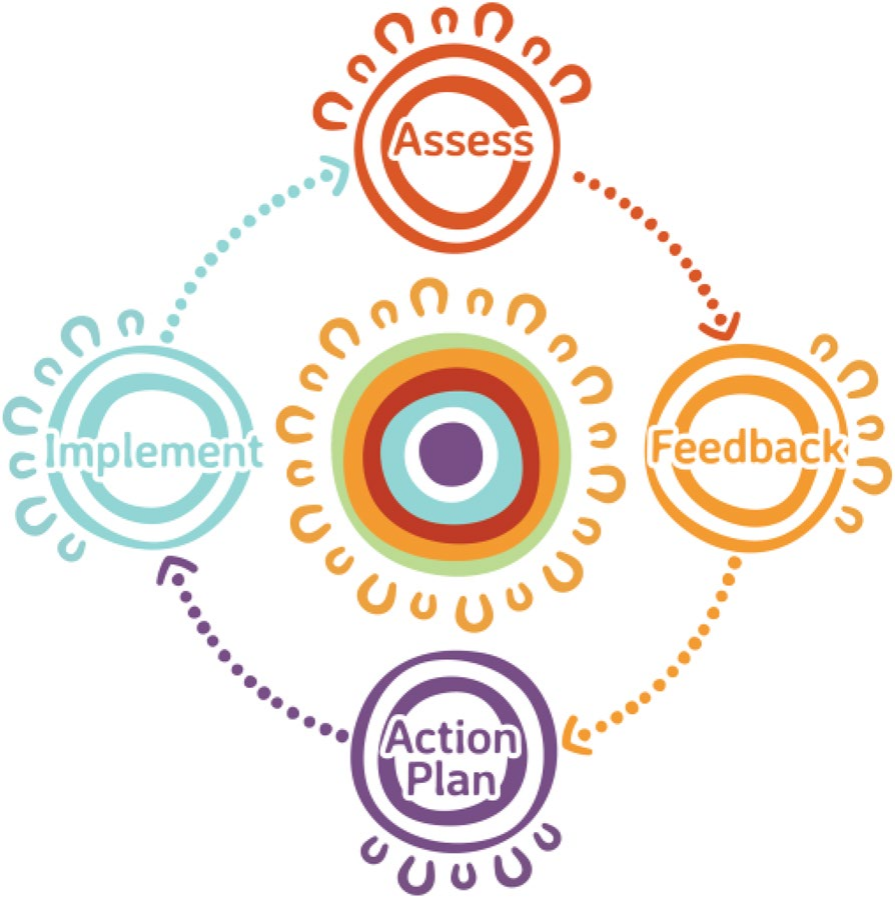
The benchmarking and continuous improvement cycle of assessment, feedback, action planning and implementation. Adapted from the PLAN-DO-COLLECT-LEARN cycle, Menzies School of Health Research [63]. This figure shows the four components of the cycle and the sharing of this information with store directors/owners as represented by the middle section of the figure. Artwork by Ngarrindjeri artist Jordan Lovegrove

The intervention comprises two full benchmarking continuous improvement cycles (one per year) with all stores participating in cycle 3 (Fig. 2). A cycle comprises four components (Fig. 3):

Control stores receive an in-store assessment of their best-practice implementation at one time each year for three years (2022, 2023, 2024) and provide weekly store sales data (52 weeks pre to 52 weeks post intervention). They are offered a full benchmarking cycle in 2024, prior to which they will continue with usual retail practice.

1. *Assessment* of i) POLICY: adoption of health-enabling best-practice store policy, ii) PRACTICE: implementation of best-practice, iii) PRICE: price of a standardised healthy diet, iv) PURCHASING: healthiness of customer purchasing; v) ENVIRONMENT SCAN: Influence of enabling/impeding environment-level factors on healthy in-store operations such as disruptions to food delivery, power outages, and infrastructure.
2. *Feedback*: Within 2-3 months of assessment, intervention stores receive a visual feedback report, with results for their Policy, Practice, Price and Purchasing indicators benchmarked against best-practice targets, all stores, and their progress over time. These reports give data-informed recommendations for performance improvement and feedback on the environmental-level factors.
3. *Action Plan*: Partners present the feedback report to community store directors (store committee/board) in a face-to-face meeting and co-design a feasible and acceptable action plan to improve performance with the ‘who’, ‘how’ and ‘when’ described. Strategies to be employed as part of the action plan may include 'No price promotions on discretionary food and drink products', 'Price discounts on fruit and vegetables', 'No discretionary food and drink products in highly visible/high traffic areas of the store including front-of-store, check-outs, end-of-aisle caps'
4. *Action Implementation*: The co-designed action plan is implemented by the store staff with assistance from the partners prior to the next assessment. Nutrition practitioners are encouraged to maintain regular communication with store staff to maintain motivation. Control stores receive an in-store assessment of their best-practice implementation at one time each year for three years (2022, 2023, 2024) and provide weekly store sales data (52 weeks pre to 52 weeks post intervention). They are offered a full benchmarking cycle in 2024, prior to which they will continue with usual retail practice.

#### Theory of change

Principles of Continuous Quality Improvement have informed our benchmarking model. This includes a focus on organizational process and systems, rather than on individuals within the system; the use of structured audit, feedback and action planning approaches; and, empowerment of stores to identify performance-standard gaps and action improvement opportunities [39, 40]. We draw on concepts from Feedback Intervention Theory (FIT) and goal-setting theory to consider the relationship between the Benchmarking intervention and the outcomes of improved best-practice policy and practice adoption and healthier food purchasing [69, 70]. FIT argues that feedback-standard gaps regulate behaviour by modifying the locus of attention, that then effects task learning and motivation [69]. It argues that people choose to eliminate the feedback-standard gap by attaining the standard when the goal is clear, when high commitment is secured for it, and when belief in eventual success is high [69, 70]. Our benchmarking model provides feedback to stores on their best-practice standard and target gap and their rank in performance with other stores. Through action planning, clear goals are set with stores to attain the best-practice standard.

#### Criteria for discontinuing or modifying allocated interventions for a given trial participant

There is no allowance for modifying allocated interventions. Participants (the store businesses) have the right to withdraw from the study at any time. Otherwise all consented intervention stores participate as planned in the benchmarking with continuous improvement. Implementation fidelity is monitored by the research team.

#### Strategies to improve adherence to intervention protocols

The research team provide training in the benchmarking intervention to nutrition practitioners and coaching as requested. Implementation is monitored using i. a resource-use survey (completed by nutrition practitioners after each benchmarking component, for the purpose of the economic evaluation), ii. documentation of action-plan summaries (using an excel spreadsheet), and, iii. logging of survey completions in REDCap. Interviews are conducted with partner representatives, store directors/owners and managers in 2023 and 2024 (following the Action Plan component of the cycle) to assess implementation and adoptability.

#### Relevant concomitant care and interventions that are permitted or prohibited during the trial

In this real-world pragmatic trial researchers do not have control over the practice of the participating store businesses. Data on relevant initiatives, programs, interventions that stores initiate independently to the benchmarking trial are collected through interview with store managers and through a resource-use survey in 2023 and 2024 that also collect usual practice activity.

#### Outcomes

Primary outcome: Change in the last six-months (July-December 2023) from baseline (July-December 2021) in free sugars from all food and drinks purchased (g sugar/MJ total energy), derived from electronic point-of-sale data.

Secondary outcomes: Change from baseline to i) last six months of 2022 and ii) last six-months of 2023 for the following purchasing outcomes derived from data of all food and drinks purchased:

- free sugars (g/MJ total energy)
- discretionary products weight (g/MJ total energy)
- core products weight purchased (g/MJ total energy)
- sodium (mg/MJ total energy)

Change from baseline (2022) in 2023 and 2024 in adoption of best-practice store policy action and practice implementation.

#### Participant timeline

For intervention stores, the benchmarking cycle involves an assessment in July/August of 2022, 2023 and 2024. Feedback reports are then provided to partners for store groups and independent stores directly between October/November of 2022, 2023 and 2024. Action plan meetings are arranged by partners with stores following receipt of feedback reports in 2022 and 2023. Store directors/owners/ management then have responsibility to implement the co-designed action plans. Action implementation occurs following action plan meetings to June of each year before assessment starts again in July/August.

For control stores, data collection occurs in July/August, 2022 (using Store Scout App) and 2023 (using Store Scout App and Healthy Diets ASAP). One full benchmarking cycle will be completed, commencing with assessment in July/August, 2024.

As shown in Table 1, weekly sales data are collected from intervention and control stores from 2021 to 2025.

**Table 1:** Summarised benchmarking performance metrics and data collection methods

Benchmark assessment area	Stores assessed	Data collector	Data collection instrument (Supplementary material)	Estimated data collection time
i) Policy: adoption of health-enabling best-practice store policy	Intervention stores (2022, 2023, 2024) Control stores in 2024	Store owner/s (for privately owned stores), store representatives or store nutritionists for stores with ALPA or Outback Stores (and with area managers for Outback Stores)	Policy Action Progress Audit e-survey: 24 three-part item survey on adoption status (full, partial, getting started, not yet started) of 21 industry- and research-informed policy actions [62]. Survey informed by research evidence, the remote food retail, government policy and health and research sectors, and BIA-Obesity, and built in REDCap [32, 64].	~ 30 min
ii) Practice: implementation of best-practice	Intervention and control stores (2022, 2023, 2024)	Trained partner nutrition practitioners/research team	Store Scout App [65]: 293 item survey on product, placement, price promotion, and product promotion in seven food/drink categories [59].	40-60 min
iii) Price: Price of standardised healthy diet	Intervention and control stores (2023/2024 only)	Trained partner nutrition practitioners/research team	Aboriginal and Torres Strait Islander Healthy Diets ASAP: on-line survey to collect food price data on 76 healthy and unhealthy food/ drink products [61].	~ 60 min
iv) Purchasing: Healthiness of customer purchasing	Intervention and control stores (2021–2025)	Trained partner nutrition practitioners/research team	Weekly sales data provided by store businesses (Product identifier, Product description, quantity sold, dollar value) [66].	Varies depending on store point of sale systems
v) Environment Scan: Level of influence of environmental-level enabling/impeding factors	Intervention and control stores (2022*, 2023, 2024) *2022 – Intervention only	Store owner/s (for privately owned stores), store representatives, or store nutritionists for stores with ALPA or Outback Stores (with or without store managers and/or area managers)	Environment scan: 26-item e-survey to collect data on the broader socio-ecological impacts on store practice. Survey informed by publicly available submissions to a Parliamentary Inquiry and the Good Food Planning Tool [67, 68]. Co-designed with stakeholders and built in REDCap	~ 15 min

#### Sample size

There are 53 eligible stores in 45 communities across the NT serviced by the partner s. We anticipate from previous trials an 85% consent rate and no withdrawals (or loss to follow up for the primary outcome). A sample of 36 communities will have 90% power to detect an ~ 3.5% lower percentage change in the primary outcome, free sugars g/MJ (SD of % change = 3%). The mean effect size of an ~ 3.5% reduction in free sugars (g/MJ) is based on our Healthy Stores 2020 trial [4]. We commenced recruitment in 2022 and recruited 32 stores (Fig. 1 and Additional Table 1).

#### Recruitment

Partner nutrition practitioners advised on and assisted with the community engagement process to invite store directors in each of their regions. Nutrition practitioners and/or the research team (where required), provided store directors/owners either directly or via their store manager(s) and/or area managers with an invitation to participate, including information about the project (short video or flyer), project information sheet and consent form. These were delivered in person or via email. Follow-up contact with the store owners/manager(s) was made by the nutrition practitioner and/or research team to arrange a meeting (face-to-face, or via videoconference) to explain the study, its purpose and consent procedure, in detail. For some stores, store managers and/or store group area managers were also involved. This was to arrange a suitable time for the meeting and to communicate study detail to Aboriginal and Torres Strait Islander store directors to ensure free and fully informed consent. Where required and within two-weeks of the meeting, a follow-up phone call was made to the store owner(s)/manager(s) by the nutrition practitioner and/or research officer to inquire on their decision. Phone contact up to three times if needed was allowed. We have found these methods for store recruitment to be acceptable to communities [4, 71].

## Methods: assignment of interventions (for controlled trials)

### Allocation

Computer generated random numbers were used to allocate stores in near equal numbers to intervention/control within the store group serviced by each partner organisation (i.e., ALPA, NT Health (Central Australia), NT Health (Top-End), Katherine West, Miwatj, Outback Stores). Two stores with ALPA and two stores serviced by Miwatj were forced as each of these store pairs were in the same community and so 15 stores were allocated to intervention and 17 to control. Allocation occurred simultaneously after the required sample of stores was reached.

### Blinding

Those analysing the outcomes will be blinded. Blinding is not possible for other members of the research team, data collectors, partner representatives and participating stores.

## Methods: data collection, management, and analysis

Data collection methods are described in Table 1. Research practice against the ‘CREATE Tool’ is reported in Table 2.

**Table 2:** Research practice considered against the CREATE Tool best-practice guidance

CREATE TOOL practice	Benchmarking for healthy remote stores in remote Aboriginal and Torres strait islander communities—research practice
1.Does the research respond to a need or priority determined by the community?	This research responds to a call made publicly through the 2020 Parliamentary Inquiry into food pricing and food security in remote Indigenous Australia, by remote Aboriginal and Torres Strait Islander food retail organisations, Aboriginal and Torres Strait Islander health services and academics for benchmarking to standardise health-enabling retail practice for the benefit of all communities [2]. It builds on the decades of collaborative and reciprocal research that Aboriginal and Torres Strait Islander communities have informed and contributed to with members of the author group, and brings together Indigenous and non-Indigenous academics and partners who have worked with remote communities for many years to address inequities in access to healthy and affordable food. It is informed specifically by a continuous improvement approach to improving food security in remote communities that was developed in 2009–2013 with four remote Aboriginal and Torres Strait Islander communities and other stakeholders [39, 40]. It seeks to ultimately strengthen the decision-making capacity of Aboriginal and Torres Strait Islander store owners/directors in tackling the devastating impact on health experienced by Aboriginal and Torres Strait Islander Peoples in remote communities from a rapid change to the food environment, especially the ready availability of energy-dense, nutrient-poor discretionary foods and beverages. This research takes a strengths-based approach to influence diet as a social determinant of health and wellbeing and in doing so aligns directly with two of the three priority research areas of the National Health and Medical Research Council Road Map 3: A strategic framework for improving Aboriginal and Torres Strait Islander Health through research and with the National Agreement Closing the Gap Priority Reform 4 “Improve and share access to data and information to enable Aboriginal and Torres Strait Islander communities make informed decisions” [72, 73].
2.Was community consultation and engagement appropriately inclusive?	Consultation in the grant writing and project planning processes occurred with several Aboriginal organisations including ACCHOs and ALPA, and government and Outback Stores. These partners then worked with the research team to inform Aboriginal and Torres Strait Islander directors and private store owners of the study and invite their participation. To uphold trustworthiness we approached Aboriginal and Torres Strait Islander store directors by presenting on the study at one of their board meetings and gave appropriate time for a collective board decision. All partners were invited to participate in the structures established for co-design of the benchmarking with continuous improvement model and research processes. Aboriginal and Torres Strait Islander community members contributed to the co-design of the Benchmarking strategy through workshops
3.Does the research have Aboriginal and Torres Strait Islander research leadership?	Three of the ten project Chief Investigators are senior Aboriginal leaders and academics. Aboriginal and Torres Strait Islander leadership is also provided through the Associate Investigator group and the partner organisations
4.Does the research have Aboriginal and Torres Strait Islander governance?	The project is designed with multiple levels of governance. The Chief Investigator group oversees a project benchmarking co-design committee that has representatives from all partner organisations, the Chief Investigator group and the research team. Four task groups comprising members from the benchmarking co-design committee feed in to the benchmarking co-design committee and have responsibility for the co-design of the benchmarking model and data collection tools and research processes. The partner organisations are the face of the research with participating stores and liaise with store directors ensuring local community protocols are respected and followed. The Chief Investigator group with the project lead guides the research team who coordinates the research activity and delivery of study milestones
5.Are local community protocols respected and followed?	We are advised by our partners and Aboriginal and Torres Strait Islander store directors on community protocols and when it is a suitable time to visit the community and/or meet with Aboriginal and Torres Strait Islander store directors. Written permits are required under Commonwealth and Territory law to enter Aboriginal land. The Northern Land Council is responsible for administering the permit system for traditional owners in the Top End, and the Central Land Council for traditional owners in Central Australia, Northern Territory. Permits are therefore obtained prior to visiting Aboriginal Land by the research team and partners. All endeavour is taken to remain flexible with the implementation of the research to ensure there is no adverse impact on other community priorities. For example, meetings will be rescheduled if this is advised by store directors and/or partner representatives We conduct our research with a commitment to the spirit and integrity of Aboriginal and Torres Strait Islander Peoples and demonstrate a high regard and respect for the welfare, rights, beliefs, perceptions, customs and cultural heritage of both the individual participants involved in our research and the community as a collective
6.Did the researchers negotiate agreements in regards to rights of access to Aboriginal and Torres Strait Islander peoples’ *existing* intellectual and cultural property? 7.Did the researchers negotiate agreements to protect Aboriginal and Torres Strait Islander peoples’ ownership of intellectual and cultural property *created* through the research?	A multi-institutional agreement (MIA) for the project has been negotiated between all participating research institutions and partners, outlining the funding allocation, each organisation’s role, contribution, and ownership/custodianship and use of background and project created intellectual property. Data remain the property of the community and store directors/owners with investigators and partners being the custodians The study consent form ensures that participants provide consent knowing that ownership of Aboriginal and Torres Strait Islander knowledge and cultural heritage is retained by participants should this be shared (although it is likely due to the nature of the project that this type of information is not shared). This is also a requirement of the Human Research Ethics Committee with oversight for this project
8.Did Aboriginal and Torres Strait Islander peoples and communities have control over the collection and management of research materials?	Data are only to be collected and used for the purpose that Aboriginal and Torres Strait Islander store directors/ owners and/or individuals have given written consent for. Consented store directors/owners and individuals have been informed of how the data will be managed. In the final year of the project a governance structure and agreed on protocol to guide future potential use of the project data and the Benchmarking model will be finalised with Aboriginal and Torres Strait Islander stakeholders
9.Was the research guided by an Indigenous research paradigm?	This research has been guided by respect for the rights of store directors/owners to be informed of best-practice health-enabling store policy and practice whilst having the right to determine what policy and practice is best for their store and community. The randomised controlled design of this research suggests a positivist paradigm has guided the research where knowledge is revealed through measurable observation and if it can’t be measured in this way than it is uncertain. However, we are guided in this research by a primary interest in how the co-designed benchmarking strategy can best serve store directors in their effort to contribute to their community’s health. If we find from the effectiveness trial that it does not have the hypothesised impact, then we will seek to understand with Aboriginal and Torres Strait Islander stakeholders how the strategy can be strengthened and what value it has. If the strategy is found to be effective and of value, then we will seek to answer with Aboriginal and Torres Strait Islander stakeholders how it can best be sustained. This research is therefore guided by the belief that there can be many truths that are based on context and peoples’ experiences and that knowledge cannot be separated from context. It also acknowledges that differences in worldviews must be recognised and listened to deeply in order to co-design a strategy that is meaningful for Aboriginal and Torres Strait Islander Peoples and is of value and does no harm to Aboriginal and Torres Strait Islander communities
10.Does the research take a strengths-based approach, acknowledging and moving beyond practices that have harmed Aboriginal and Torres Strait peoples in the past?	This research directly respects Aboriginal and Torres Strait Islander store directors as policy-makers for their store and experts on what works best for their store and community. It also respects partner nutrition practitioners (and their supports) as having the relationships and on-the-ground contextual knowledge needed for delivery of the benchmarking model. The research respects these knowledges and capabilities and is designed to harness and assist these to ultimately support implementation of incremental continuous improvement towards health-enabling best-practice policy and practice
11.Did the researchers plan to and translate the findings into sustainable changes in policy and/or practice?	The research directly plans to delineate how the co-designed benchmarking strategy can be embedded in to ongoing service delivery and policy
12.Did the research benefit the participants and Aboriginal and Torres Strait Islander communities?	This research aims to directly support store directors to adopt best-practice policy and practice for the health of the communities they serve. It will further position Aboriginal and Torres Strait Islander store directors, other community leaders, researchers and health services to advocate for equitable and health-enabling policy and practice for Aboriginal and Torres Strait Islander communities. Wide-scale implementation of best-practice policy and practice across remote stores is likely to significantly impact the excessive burden of diet-related preventable chronic conditions that are disproportionately experienced by Aboriginal and Torres Strait Islander Peoples living in remote communities
13.Did the research demonstrate capacity strengthening for Aboriginal and Torres Strait Islander individuals?	We anticipate that this research in many cases will strengthen store director/owner—nutrition practitioner relationships and strengthen the decision-making capacity of Aboriginal and Torres Strait Islander store directors through the co-design of action plans that respect the needs and aspirations of the store
14.Did everyone involved in the research have opportunities to learn from each other?	There is a strong focus with the benchmarking model on shared learning and for Indigenous and non-Indigenous researchers and partners to have opportunity to learn from each other. The co-design structures particularly foster shared-learning between practitioners, academics, health policy makers and Indigenous and non-Indigenous Peoples. Research dissemination activities including journal and conference publication and this publication protocol encourages all who have had input in to the research to contribute and celebrate in its achievements

### Data management

Benchmarking assessments (as described in Table 1) are completed through use of an App (Store Scout App, managed by Monash University), a web portal (Aboriginal and Torres Strait Islander Healthy Diets ASAP, managed by University of Queensland) and purpose-built REDCap e-surveys (i.e., Policy Action Progress Audit, Environment Scan, resource-use surveys managed by Monash University and available on request). Trained assessors can access coaching in use of the data collection tools. Data stored on the Store Scout App and the Healthy Diets ASAP web-portal are protected with a designated user-ID and password. Users can view and edit the data they collect only.

REDCap survey data are available to specified users only with multi-factor authentication login. Individual survey links are sent to specific respondents via email with those respondents only able to view, enter and edit their own individual survey data.

Sales data are sent electronically from each of the stores, or from the store group (where relevant) to Monash University via email attachment or a password protected secure transfer file (as chosen by stores).

All data are kept secure at Monash University in a password protected directory, a shared Google drive, and in the Benchmarking for Healthy Stores database purposely designed for the study. All have designated user restrictions.

### Analysis and statistical methods

The data source for analysis of the primary outcome and some secondary outcomes is weekly sales reports provided by the stores. Unique store products are linked, using rule-based algorithms, machine learning and human verification, to nutrient data derived from the Australian Food, Supplement, and Nutrient Database, with discretionary food flagged from the Discretionary Food List developed by the Australian Bureau of Statistics [74, 75]. We use built-in, reliable data checking processes developed over the last decade in our research to assess and quantify the nutritional impact of sales.

Following data aggregation, the outcomes will be log-transformed. The main analysis for an outcome will be a simple ANCOVA analysis using sales data from July to December 2021 and 2023, and randomisation group for the primary outcome.

We anticipate that the impact of the intervention will be both immediate and gradual. In stores that receive the benchmarking intervention, we estimate an immediate 3.5% reduction in free sugars to energy purchased as a result of food retail practice change. A reduction in free sugars to energy at this level from the baseline shown in the Healthy Stores 2020 trial has been shown to be the equivalent to an approximate 10% lower risk for cardiovascular disease mortality [4]. We then expect a more gradual effect at the broader remote store level post-intervention as the benchmarking model is scaled up into policy and practice by different jurisdictions for the benefit of all remote communities, if found to be adoptable and effective.

Implementation fidelity will be assessed according to i) proportion of partner organisation representatives completing training; and, ii) annual benchmarking cycles fully completed in specified timeframes. Data will be collected on action plans and the resource input to their implementation.

Change in store best-practice policy action adoption and practice implementation with time will be described and used to interpret results of primary and secondary outcomes. Food price and Environment Scan survey results will be described for context.

Policy Action Assessment: Proportion of policy actions by adoption status (fully adopted, partially adopted, getting started, not yet started) for 2022, 2023 and 2024 for each intervention store will be calculated and any differences with time described.

Practice assessment: An overall score out of 100 is derived automatically by the Store Scout App following each store assessment in 2022, 2023 and 2024 in addition to a score out of 100 for each food/drink category. Differences in total and category scores with time for intervention stores and between intervention and control stores will be described.

## Methods: monitoring

The research investigator group meets bi-monthly and oversees decisions on study design and protocol, milestones, ethics and governance, data monitoring, trial conduct, budget and research dissemination. A Benchmarking Co-Design committee and four task groups inform the benchmarking with continuous improvement model build and approach. Adverse events or unintended effects of trial conduct or trial interventions spontaneously raised by partners and/or participants are reported to the research investigator group.

## Ethics and Dissemination

### Research ethics approval

The Benchmarking for Healthy Stores in Aboriginal and Torres Strait Islander communities project has approval from the Miwatj Health Board, Katherine West Health Board, Sunrise Health Board, NT Health Research Governance, the Northern Territory Health and Menzies School of Health Research Human Research Ethics Committee and Aboriginal sub-committee [HREC 2021–4212], the University of Queensland Human Research Ethics Committee [HREC 2022/HE001158], Curtin University Human Research Ethics Committee [HREC 2023–0070] and is registered with Monash University [HREC 2022–30761-73457]. Protocol amendments that may add additional risk to the store participants are communicated to stores prior to seeking approval from the HREC.

### Consent

Written consent is required for participating stores. The consent procedure involves store directors/ owners providing consent for the participation of their store separate to consent to be interviewed and/or participate in the project workshops. Participants provide consent knowing how their data will be managed and their right to have their data withdrawn at any time without prejudice. Informed consent was provided by all participating stores. Additional consent is sought for use of participant data in ancillary studies that may arise.

### Confidentiality

Stores will only be named in public if all participating store businesses specifically request to be named.

### Declaration of interests

The authors declare no conflict of interests.

### Access to data

Each intervention store and their supporting nutrition practitioner have access to hard-copy reports for their store only, including the policy, practice, price, purchasing and environment scan assessments. User ID and passwords for data collection tools are administered and managed by Monash University and UQ. Research investigators have access to the final datasets. No third party has access to data unless ethics approval and permission from the participating stores is granted.

### Dissemination policy

Following demonstration of effectiveness, we will utilise rapid translation strategies we have used previously. This includes regular stakeholder communication, timely feedback of preliminary results to store directors/owners, use of video to communicate research aims and findings to a broad audience, and workshops to finalise the benchmarking model and determine its embedding int service delivery and policy and its governance. In addition, this study sits within and will harness the translation capabilities and extensive national and international networks of INFORMAS and The Centres of Research Excellence in Food Retail Environments for Health (RE-FRESH) and in Strengthening Systems for Indigenous Health Care Equity [76, 77]. Authorship of publications originating from the Benchmarking for Healthy Stores project is to align with authorship guidelines and national ethical guidelines including the NHMRC Ethical conduct in research with Aboriginal and Torres Strait Islander Peoples and communities: Guidelines for researchers and stakeholders [78]; and the AIATSIS Code of Ethics. for Aboriginal and Torres Strait Islander Research [79]. Authorship must recognise, acknowledge and value the significant contribution to the Benchmarking for Healthy Stores Project of Aboriginal and Torres Strait Islander Peoples knowledges and involvement.

## Discussion

This research will result in a benchmarking model with a proposed governance, workforce and data management structure co-designed with key stakeholders including Aboriginal and Torres Strait Islander community leaders, Aboriginal and Torres Strait Islander store directors, remote food retailers, nutrition practitioners, government policy-makers and academics for its future use.

Its use in the long-term has potential to equip Aboriginal and Torres Strait Islander communities, store directors, retailers, health services, and government policy-makers with knowledge and skills of health-enabling best-practice policy and retail practice. It provides timely information for communities to monitor and strengthen the healthiness of their store and evaluate the impact of change in policy and practice on the healthiness of food and drinks purchased. This research responds to the bold initiatives remote stores are taking to further enable Aboriginal and Torres Strait Islander Peoples make health-enabling evidence-informed policy for their stores and communities.

This novel intervention has potential to support Aboriginal and Torres Strait Islander communities’ impact significantly on reducing the risk of diet-related preventable chronic disease experienced. Diet is one of the leading risk factors for chronic disease worldwide [20]. Changes in store policy and practice for improved population-level diet can benefit the health of all individuals and future generations. Noted though, is that store health-enabling policy and practice can be strengthened with Aboriginal and Torres Strait Islander store directors, however, there are limits to what stores can voluntarily achieve without government intervention to address inequities in food cost in remote communities and regulate the availability and promotion of highly processed unhealthy food and drinks.

### Supplementary Information

Supplementary Material 1.

## Data Availability

Data collection surveys and consent procedures are available on request. Data are only available to a third party with reasonable written request to the corresponding author and approval received from the project Chief Investigators, Northern Territory Health and Menzies School of Health Research HREC, participating stores and partner organisations.

## Abbreviations

ACCHOs: Aboriginal Community Controlled Organisations
ALPA: Arnhem Land Progress Aboriginal Corporation
AMSANT: Aboriginal Medical Services Alliance Northern Territory
ATNI: Access to Nutrition Initiative
CREATE Critical Appraisal Tool: The Centre of Research Excellence in Aboriginal Chronic Disease Knowledge Translation and Exchange
FIT: Feedback Intervention Theory
INFORMAS: International Network for Food and Obesity/non-communicable diseases Research, Monitoring and Action Support
Katherine West: Katherine West Health Board Aboriginal Corporation
Miwatj: Miwatj Health Aboriginal Corporation
NT Health: Northern Territory Health
NT: Northern Territory
Sunrise: Sunrise Health Service Aboriginal Corporation
