# Supplementary material for: Benchmarking for healthy food stores: protocol for a randomised controlled trial with remote Aboriginal and Torres Strait Islander communities in Australia to enhance adoption of health-enabling store policy and practice
Source: BMC Public Health. 2024 Jul 5;24:1790. doi: 10.1186/s12889-024-19277-0 (PMC11229494; doi:10.1186/s12889-024-19277-0)
Supplement: Supplementary file 1 — Supplementary Material 1. [file 12889_2024_19277_MOESM1_ESM.doc]

Additional Table 1. Schedule of enrolment, intervention, and assessments

|  |  | **STUDY PERIOD** | | | | | | |
| --- | --- | --- | --- | --- | --- | --- | --- | --- |
|  |  | **ENROLMENT** | **ALLOCATION** | **POST ALLOCATION** | | | **CLOSE-OUT** | |
| **TIME POINTS** |  | **-t_1_** | **0** | **t_0*_**  ***July-Dec 21*** | **t_1_**  ***July-Dec 22*** | **t_2_**  ***July-Dec 23*** | | **t_3_**  ***Jul-Dec 24*** |
| **ENROLMENT** | **Eligibility screen** | X |  |  |  |  | |  |
|  | **Invited** | X |  |  |  |  | |  |
|  | **Informed consent** | X |  |  |  |  | |  |
| **ALLOCATION** |  |  | X |  |  |  | |  |
| **INTERVENTION** | **Intervention – Benchmarking model** |  |  |  |  |  | |  |
|  | **Control – Usual practice^≠^** |  |  |  |  |  | |  |
| **ASSESSMENTS^¥^** | ***Free sugars (g/MJ total energy) from all food and drinks purchased^€^*** |  |  | X | X | X | |  |
|  | ***Total weight of discretionary products sold per MJ of all products sold (g/MJ)*** |  |  | X | X | X | |  |
|  | ***Core products (g/MJ) ie total gram weight of core products sold per MJ of energy from all products sold*** |  |  | X | X | X | |  |
|  | ***Total sodium (mg/MJ) of all products sold*** |  |  | X | X | X | |  |
|  |  |  |  |  | ***t_0_***  ***July/Aug 2022*** | ***t_1_***  ***July/Aug 2023*** | | ***t_2_***  ***July/Aug 2024*** |
|  | ***Policy^β^*** |  |  |  | X | X | | X |
|  | ***Practice*** |  |  |  | X | X | | X |
|  | ***Price*** |  |  |  | - | X | | X |
|  | ***Environment Scan^±^*** |  |  |  | X | X | | X |

**^*^**t_0_=baseline

**^≠^**Control stores receive intervention – benchmarking model July – Dec 2024.

^¥^ Purchasing data collected to Dec 2025 for a post-trial evaluation

^€^Primary outcome is assessed at t_0_ baseline and t_2_

***^β^***Policy measures collected for stores allocated to intervention only

***±***Environment Scan measures collected in 2022 for intervention allocated stores only and for all stores in 2023 and 2024.
